# A tricky gastric lesion diagnosed by small-bowel capsule endoscopy

**DOI:** 10.1055/a-2208-2500

**Published:** 2023-12-05

**Authors:** Willian Ferreira Igi, Elen Bruna Teixeira Boulhosa, Magda Priscila Cardoso Afonso, Fernando Lander Mota

**Affiliations:** 1 Endoscopy Unit, Advanced Digestive Endoscopy Center from Rondonia, Porto Velho, Brazil; 2 67766 Endoscopy Unit, Hospital de Amor, Barretos, Brazil; 3 468933 Medicine Department, FIMCA Curso de Medicina, Porto Velho, Brazil; 4 Endoscopy Unit, Hospital Sírio-Libanês, São Paulo, Brazil

Small-bowel capsule endoscopy (SBCE) is the first-line method for investigating overt gastrointestinal bleeding (OGB), ideally within 48 hours after the episode 1 . However, SBCE can also evaluate lesions outside the small bowel, even though that is not its original purpose. This is extremely important when considering the prevalence of lesions outside the small bowel that are missed by conventional esophagogastroduodenoscopy (EGD) or push enteroscopy, a rate that ranges from 3.5% to more than 30% 2 .

We report on a 70-year-old woman who was referred for evaluation using SBCE following recurrent episodes of OGB. She had already undergone two EGDs and a colonoscopy, neither of which identified the source of bleeding. At the time of the current examination, she presented with melena.

After swallowing the device, the capsule endoscope quickly reached the stomach, where it was possible to visualize hematinic residues ( Fig. 1 ). An EGD was subsequently performed and identified a Dieulafoy’s lesion along the anterior wall of the gastric body. A hemostatic clip was placed ( Fig. 2 ). The bleeding stopped immediately, and the patient was discharged after 2 days.

**Fig. 1:**
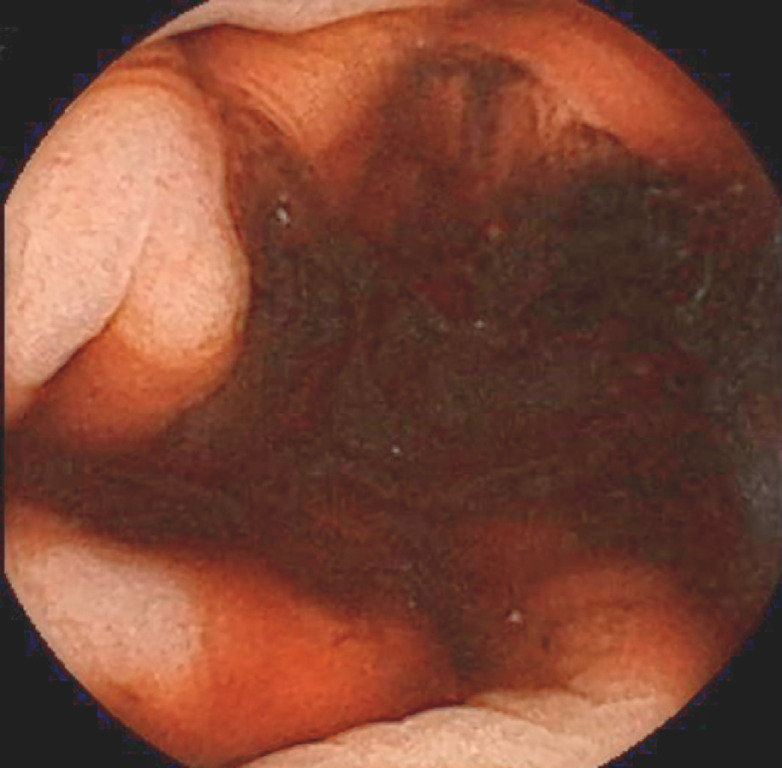
Small-bowel capsule endoscopy showed hematinic residues in the stomach.

**Fig. 2:**
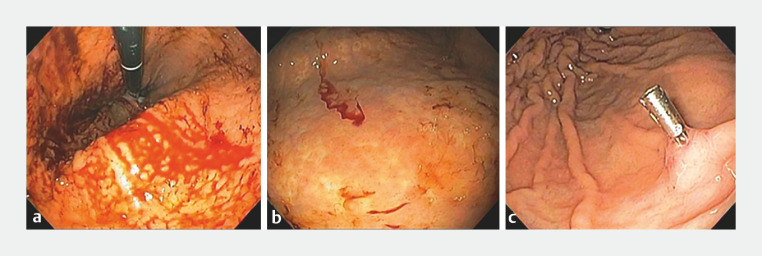
Findings on esophagogastroduodenoscopy. **a** Actively bleeding lesion in the gastric body. **b** Dieulafoy’s lesion visualized after lavage. **c** Final appearance after hemostasis with a hemostatic clip.

Subsequent analysis of the SBCE allowed the visualization of active bleeding in the stomach. Video 1 shows the diagnostic steps and treatment of the Dieulafoy’s lesion.

Dieulafoy’s lesion found on small-bowel capsule endoscopy and treated during esophagogastroduodenoscopy.Video 1

Reading of an SBCE examination should include prereading, landmarking, findings and clip selection, and reporting, as well as evaluating other segments beyond the small bowel 3 . However, this case demonstrates the need to observe the capsule’s real-time display, if available, in patients under investigation for OGB. Dieulafoy’s lesion, as presented here, is a submucosal vascular lesion identified endoscopically as a bleeding point not associated with erosions or ulcers, making its endoscopic diagnosis challenging 4 .

Endoscopy_UCTN_Code_TTT_1AP_2AB

## Notes

Endoscopy E-Videos https://eref.thieme.de/e-videos
*E-Videos* is an open access online section of the journal *Endoscopy* , reporting on interesting cases and new techniques in gastroenterological endoscopy. All papers include a high-quality video and are published with a Creative Commons CC-BY license. Endoscopy E-Videos qualify for HINARI discounts and waivers and eligibility is automatically checked during the submission process. We grant 100% waivers to articles whose corresponding authors are based in Group A countries and 50% waivers to those who are based in Group B countries as classified by Research4Life (see: https://www.research4life.org/access/eligibility/ ). This section has its own submission website at https://mc.manuscriptcentral.com/e-videos .
